# In Search of Novel Degradation-Resistant Monomers for Adhesive Dentistry: A Systematic Review and Meta-Analysis

**DOI:** 10.3390/biomedicines10123104

**Published:** 2022-12-01

**Authors:** Vlasta Mocharko, Paulo Mascarenhas, Ana Mano Azul, António H. S. Delgado

**Affiliations:** 1 Instituto Universitário Egas Moniz (IUEM), Monte de Caparica, 2829-511 Almada, Portugal; 2 Centro de Investigação Interdisciplinar Egas Moniz (CiiEM), Monte de Caparica, 2829-511 Almada, Portugal; 3 Division of Biomaterials and Tissue Engineering, UCL Eastman Dental Institute, University College London, Royal Free Hospital, UCL Medical School, Rowland Hill Street, Hampstead NW3 2PF, UK

**Keywords:** acrylate monomers, degradation-resistant, dental adhesion, dentin bonding agents, hybrid layer, hydrolysis: hydrolytically resistant, resin-based materials

## Abstract

This study aimed to assess whether degradation-resistant monomers included in experimental dental adhesives can improve long-term bond strength compared to conventional monomers. This study followed the latest PRISMA guidance (2020). The search for the systematic review was carried out in four electronic databases: PubMed/Medline, Scopus, SciELO and EMBASE, without restrictions on the year of publication and language. The last screening was conducted in July 2022. Interventions included were in vitro studies on experimental dental adhesives that tested short-term and long-term bond strength, but also water sorption and solubility data when available, in extracted human molars. Meta-analyses were performed using Rstudio v1.4.1106. A summary table analyzing the individual risk of bias was generated using the recent RoBDEMAT tool. Of the 177 potentially eligible studies, a total of 7 studies were included. Experimental monomers with acrylamides or methacrylamide–acrylamide hybrids in their composition showed better results of aged bond strength when compared to methacrylate controls (*p* < 0.05). The experimental monomers found better sorption and solubility compared to controls and were significantly different (*p* < 0.001). It is possible to achieve hydrolytically resistant formulations by adding novel experimental monomers, with chemical structures that bring benefit to degradation mechanisms.

## 1. Introduction

Dental adhesives have undergone major changes in their chemistry over the last 60 years, with the aim of creating increasingly simple procedures but with stable and lasting bonds, especially to dentin, a complex and intricate substrate [1,2]. On the contrary, bonding to enamel has remained relatively simple and effective since the discovery of the etching effect and its benefit in 1955 [3]. Currently, adhesion to dental substrates can be performed using etch-and-rinse adhesives that include a separate acid-etching step (commercialized in two or three steps), self-etch adhesives (one or two steps) or universal adhesives, which are all-in-one adhesives that allow the clinician to choose the preferred bonding strategy [4,5].

Despite the enormous evolution of dental adhesives, certain challenges remain to be solved. Specifically, the formation of the hybrid layer in dentin and its longevity is repeatedly questioned [6,7]. The degradation of the hybrid layer occurs by two interdependent and cyclic mechanisms: hydrolytic degradation, causing the dissolution of collagen fibrils and/or loss of integrity of the polymeric network; and the enzymatic degradation of the organic content by endogenous endopeptidases [8]. Salivary esterases may also contribute to the biodegradation of polymers [9]. On one hand, although the presence of water in dentin is essential to maintain the structure of the collagen network expanded, required for the resin monomers to infiltrate, when it is present in excess, it can cause separation between hydrophobic and hydrophilic monomers, creating voids, gaps and bubbles at the adhesive interface, further contributing to hydrolytic degradation [10]. On the other hand, there is the enzymatic degradation, extensively described in the literature, that occurs due to the presence of endogenous enzymes, such as metalloproteinases (MMPs) and cysteine cathepsins (CCs), which hydrolyze the organic matrix of demineralized dentin, causing deterioration and disarray in the network of collagen [11]. Both phenomena make the interface highly susceptible to leakage and bacterial ingress, leading to an inevitable degradation of the resin–dentin interface. The adhesive interface is therefore subject to chemical and mechanical degradation [12,13].

The hydrolysis of resin-based materials and their plasticization occur due to exposure to water or oral fluids [14] and can also be catalyzed by enzymes released by bacteria [15] or host-derived [16]. In addition to mechanical stress, common oscillations in the intraoral temperature can also affect the integrity of the interface [17]. All these factors are responsible for a well-documented decline in the bond strength of adhesive materials, ultimately risking the lifetime of the restoration. Because of these issues, research has been carried out to develop new strategies, such as the synthesis of new monomers with alternative chemical groups capable of resisting the degradation promoted in the intraoral environment [18,19,20]. However, to date, a synthesis of this relevant information has not yet been made. There is still little information about the best monomers under study, which show less susceptibility to degradation, as well as meta-analytical data related to this topic [21]. Such data will allow the formulation of materials with greater longevity and clinical success rates. That said, it is necessary to synthesize this information and compare the different existing strategies to guide clinical decisions based on the formulation of new dental materials.

Hence, the aim of this study was to systematically review the literature for in vitro studies which evaluated immediate vs. long-term bond strength measurement in restorative procedures using adhesive materials that contain degradation-resistant monomers. These monomers would be designed to be used in dentin and would be compared to control materials with conventional monomers. The hypothesis tested was if degradation-resistant monomers, included in experimental dental adhesives, can improve the bond strength after aging, in comparison to conventional monomers.

## 2. Materials and Methods

The present systematic review was planned and undertaken in conformity to the latest PRISMA 2020 statement guidelines [22]. The protocol for this study was submitted to the PROSPERO international prospective register of systematic reviews and registered under no. CRD42022304393 “https://www.crd.york.ac.uk/PROSPERO/display_record.php?RecordID=304393 (Accessed on 5 November 2022)”. The research question was: Are degradation-resistant monomers, included in experimental adhesives, able to improve the bond strength after aging, in comparison to conventional monomers?

### 2.1. Literature Search, Inclusion and Exclusion Criteria

The search period started on 28 June 2022, and the last search was carried out on 12 July 2022. The bibliographic databases used for the electronic search were PubMed/Medline, Scopus, EMBASE and SciELO. In addition, the reference lists of eligible primary studies were manually searched. An unrestricted publication period was chosen with no language restrictions. An individualized search strategy was developed for each database (Table 1). After paper screening, all studies were imported into Mendeley Desktop 1.19.8 (Mendeley Ltd., London, UK) to remove duplicates.

#### 2.1.1. Inclusion Criteria

The inclusion criteria were in vitro studies that included new experimental monomers, in adhesive materials, and that tested short- and long-term bond strength and water sorption. These laboratory studies had to be performed in permanent or deciduous human molars. The interventions included were adhesive restorations, in dentin, which were testing a new monomer developed as a degradation-resistant strategy. Only studies that had a control group of comparison were included (with conventional adhesive systems or experimental adhesives with conventional monomers).

#### 2.1.2. Exclusion Criteria

All non-laboratory studies, or studies that only tested the immediate bond strength, that used bovine teeth or did not include control groups were excluded. Additionally, if any of the studies did not perform tensile, microtensile, shear or microshear bond strength measurements, they were also excluded from the synthesis.

### 2.2. Data Extraction

Two reviewers (V.M. and A.D.), working independently, screened the data extracted from each database by applying the inclusion and exclusion criteria set out a priori. The researchers were blinded to individual decisions. In a final meeting, both reviewers converged their results. When a reviewer considered a paper potentially eligible, the full text was retrieved and analyzed. This happened independently and in duplicate. Full texts were exported, and a database for information retrieval was created in Microsoft Excel 365 v.17 for Microsoft spreadsheets (Microsoft, Redmond, WA, USA). Differences were resolved by consensus. This was subject to approval by all team members. It was initially tested and used thereafter.

The data extracted from each eligible primary study include authors/publication date, intervention and objective, sample size, experimental groups involved, materials used, bond strength results (immediate and aged results), bond strength test type, aging type and duration and the main conclusions.

For the articles that presented the information in graph formatting, the mean and standard deviation was calculated using WebPlotDigitizer 4.5 software.

### 2.3. Quality Assessment

Risk of bias (RoB) measurement was conducted by two reviewers, again working independently. Consensus was resolved by seeking a third review team member. The risk of bias tool used in this study was the recently developed RoBDEMAT tool [23]. This tool includes the following sources of bias: bias in planning and allocation (proper randomization and sample size calculation), bias in sample/specimen preparation, bias in outcome assessment and bias in data treatment and outcome reporting. A table summarizing the RoB results was made and included in the SR. Each signaling question was answered as “sufficiently reported”, “insufficiently reported”, “not reported” or “not applicable”. An overall summary RoB score was not produced as it was kept as a simple checklist. 

### 2.4. Meta-Analysis

To enable a quick and direct comparison between the experimental monomers and the resulting adhesive formulations, a meta-analysis of quantitative results, such as immediate and aged bond strength, but also water sorption and solubility (when data were available), was planned. All meta-analytical procedures related to effect size calculations and random effects modeling (restricted maximum likelihood method) were conducted by fitting multivariate meta-analysis using the R tools under the “metafor” package [24] in Rstudio 1.4.1106. The associated confidence intervals were adjusted for the within- and between-study treatment correlations by including an unstructured covariance matrix in the multivariate model. This model approach was followed because the data structure, where treatments were almost always exclusive to a single study, formed disconnected sub-nets, not allowing the traditional network meta-analysis to be run. The treatment pairwise network graph was built in the Metainsight online platform [25], while related forest plots were designed in Microsoft Excel 365 v. 16 for Microsoft (Microsoft, Redmond, WA, USA). For each meta-analysis, the Z test evaluated each treatment effect’s significance, while Tukey’s HSD (honestly significant difference) test evaluated the differences between treatment effects. Differences were considered statistically significant at *p* < 0.05. The initial mean bond strength results (in μTBS) and the treatment materials’ solubility and water adsorption capacity, with the associated uncertainties, were used to fit the meta-analysis, where multivariate mean estimates were obtained from the direct evidence. To obtain the differential bond strength results, the aged results obtained at the follow-up period of 6 months or by thermocycling aging were subtracted from the initial immediate values at 24 h.

## 3. Results

### 3.1. Search Strategy

**Figure 1:**
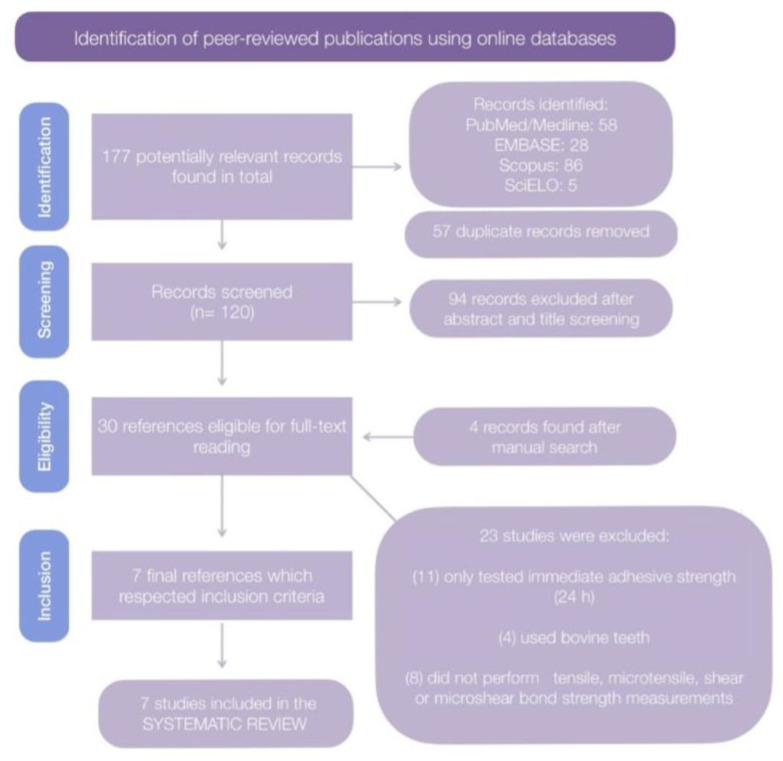
Flowchart in compliance with the PRISMA statement guidelines, showing the steps followed in each stage of the systematic review. Out of 177 potentially relevant papers, after screening, eligibility, and inclusion, 7 remained.

A total of 177 potentially relevant records were found in all of the databases. A flowchart was made, outlining the study selection process according to the PRISMA statement [22] (Figure 1).

After removing the duplicates, 120 articles were selected for the initial screening. After reading the title and abstract, a total of 30 studies were eligible for full-text reading, which included 4 studies retrieved in the manual search [26,27,28,29]. Out of these 30, 23 studies were excluded, since 11 only tested immediate bond strength (24 h), 4 used bovine teeth and 8 did not perform tensile, microtensile, shear or microshear bond strength measurements. Thus, a total of 7 studies were included in the systematic review and meta-analysis.

### 3.2. Systematic Review

All seven of the studies presented higher bond strengths of the experimental adhesives that were formulated, compared to the control groups. Three studies had lower water sorption and solubility [26,30,31], whilst one study had higher water sorption results [28]. The aging method varied from water storage (in distilled water) to thermocycling (5000 cycles or 30,000 cycles) (Table 2). 

### 3.3. RoB Analysis of the Studies

Risk of bias and the factors considered for the analysis are presented in Table 3. For the seven studies included in this SR/MA, a control group was present and reported in all, while no sample size calculation was found in any of them. Further, regarding bias in planning and allocation, the correct randomization of samples was not reported in five out of seven studies [26,28,31,32,33]. Blinding of the testing operators was not reported in any of the studies. Doubts concerning sufficient reporting of the statistical analysis were raised in four of the studies [26,28,32,34], while reporting of all expected outcomes was insufficient in the studies by Fugolin et al., (2020, 2021), Yu et al., (2021) and in Zhao et al. (2022), who reported bond strength without showing failure mode analyses data. 

### 3.4. Meta-Analyses

Of the seven studies selected for the systematic review, four of them [26,28,31,32] comprised 6 months of aging in distilled water, and two [30,34] applied 5000 thermocycling cycles in an artificial aging model. One of the studies employed artificial aging using 30,000 thermocycling cycles. This was not included in the meta-analyses as there were no other comparisons [33].

**Figure 2:**
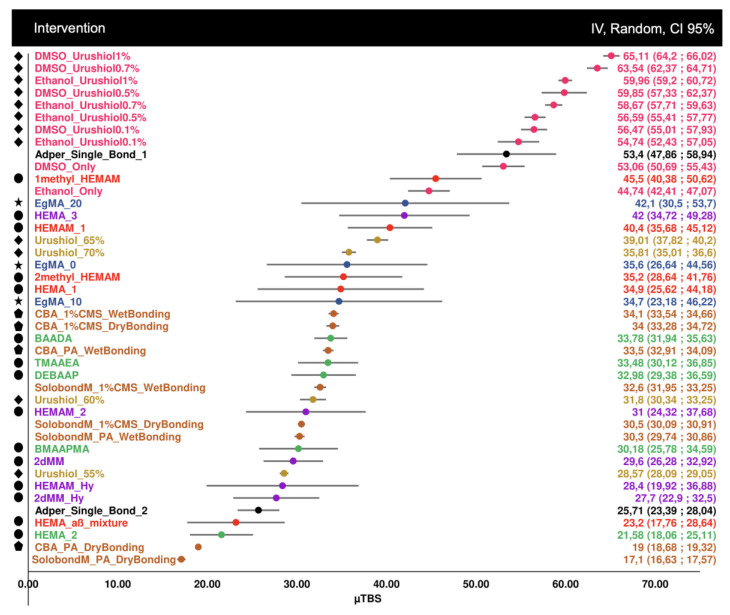
Forest plot of the immediate microtensile bond strength, including 7 distinct studies with different strategies and 3 commercial controls, which do not have any symbol, purposefully—Adper Single Bond 1, Adper Single Bond 2 and Solobond M. Symbol legend: **★** eugenyl methacrylate derivatives; ● methacrylamide derivatives; ◆ urushiol derivatives; ⬟ CBA monomer derivatives.

For the first analysis (immediate bond strength), 42 datasets were considered, which in total belonged to seven different studies (Figure 2). These groups varied in bond strengths between 17 and 65 MPa, with urushiol monomers at higher concentrations achieving higher bond strengths, but also the methacrylamide monomers, when compared to the control groups.

**Figure 3:**
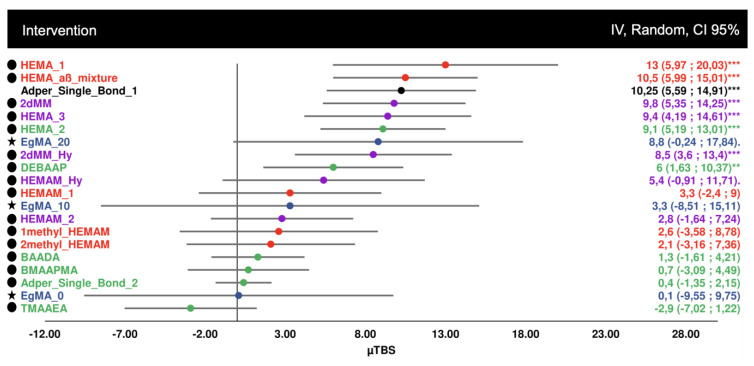
Forest plot of the bond strength difference after real aging in distilled water (6 months), by subtracting the final from the initial bond strength value. Symbol legend: **★** eugenyl methacrylate derivatives; ● methacrylamide derivatives. Statistical significance (difference to 0) is shown in asterisks, where * *p* ≤ 0.05 ** *p* ≤ 0.01 *** *p* ≤ 0.001.

For the second analysis, comparing bond strength after 6 months of aging, by subtracting the initial immediate bond strength values, 20 datasets were considered, with four studies included (Figure 3). Wider confidence intervals can be seen, associated with uncertainty, although it is possible to ascertain that the acrylamide monomers showed much smaller bond strength differences, after aging, compared to the methacrylate controls. Monomers such as HEMA derivatives showed unstable aged bond strength. 

**Figure 4:**
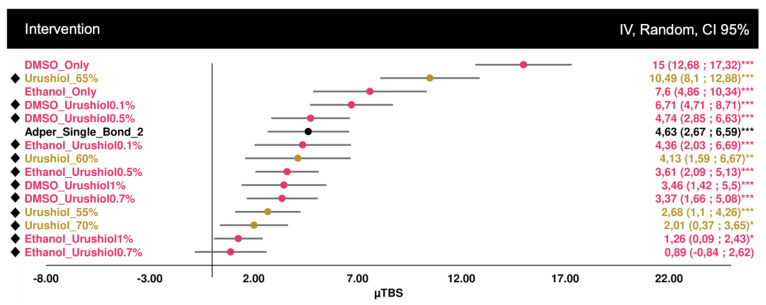
Forest plot of the bond strength subsequent to artificial aging (thermocycling—5000 cycles) subtracted from the initial bond strength values. Symbol legend: ◆ urushiol derivatives. Statistical significance (difference to 0) is shown in asterisks, where * *p* ≤ 0.05 ** *p* ≤ 0.01 *** *p* ≤ 0.001.

For the third analysis (artificial aging vs. immediate bond strength), 15 datasets were considered, although two studies were included (Figure 4). The group Ethanol_Urushiol0.7% was the only group which did not show a statistically significant decrease to the control (marked as zero), representing the immediate bond strength data. DMSO alone, without any added hydrolytically stable monomer, showed the worst performance, significantly different to all others, while the control group Adper Single Bond 2 was similar to most treatments, except to urushiol at 0.7 and 1% combined with ethanol, which superseded it.

**Figure 5:**
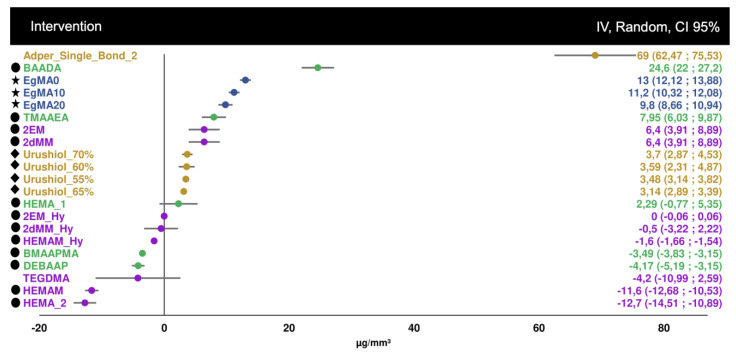
Forest plot that includes data of the water solubility measured in 4 individual studies. Symbol legend: **★** eugenyl methacrylate derivatives; ● methacrylamide derivatives.

**Figure 6:**
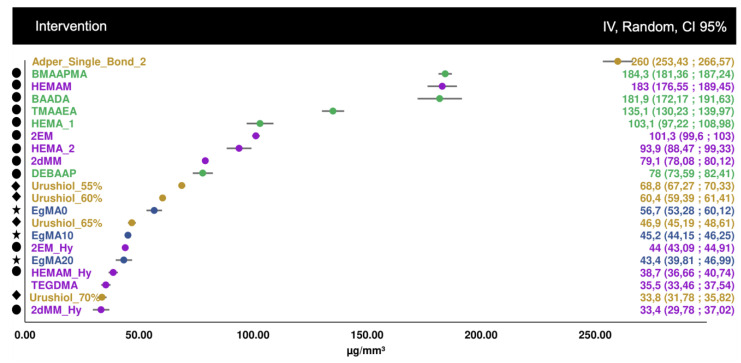
Forest plot that includes data of the water sorption measured in 4 individual studies. Symbol legend: **★** eugenyl methacrylate derivatives; ● methacrylamide derivatives.

A separate analysis was performed for the water solubility (Wsl) (Figure 5) and water sorption (Wsp) (Figure 6), as the aim of this work was also to report data that would help evaluate the hydrolytic stability of experimental adhesive formulations. For both analyses, four studies and 21 datasets were included. The Wsp and Wsl of the only commercial adhesive included in these datasets reached much higher and significantly different values to that of the experimental group formulations (*p* < 0.001), denoting that the experimentals have markedly less sorption and solubility.

**Figure 7:**
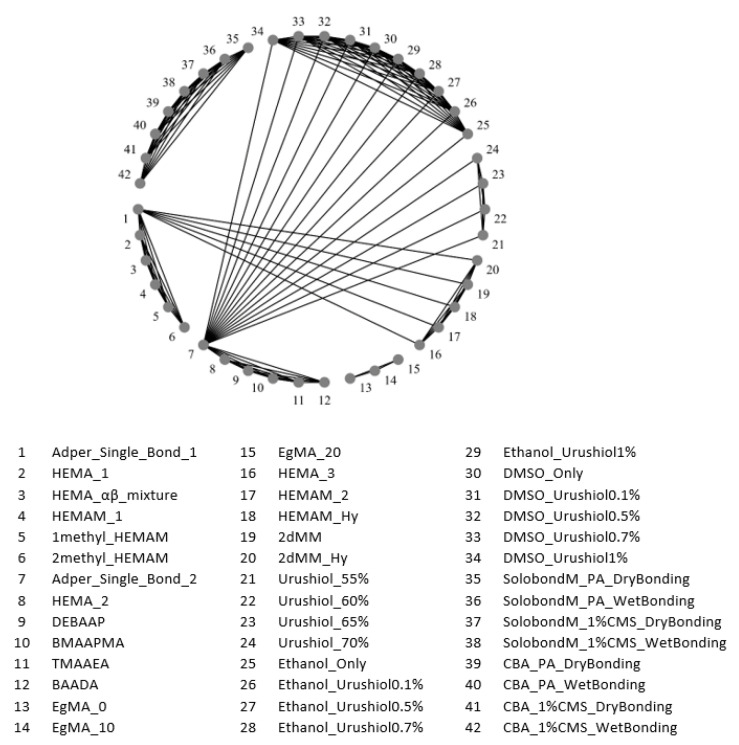
Graph of treatment network in pairs of immediate bond strength.

It was not possible to perform a meta-analysis in a traditional network, since the treatments were almost always exclusive to a single study, thus forming disconnected sub-networks, as shown in Figure 7. This figure shows the different treatments framed in the respective studies. The dimensions of the spheres are proportional to the sizes of the samples, thus making it possible to assess which treatments have results based on larger and more representative samples, and which are represented by smaller samples.

## 4. Discussion

The present systematic review and meta-analyses analyzed the data from in vitro studies that evaluated immediate vs. long-term bond strength measurement (6 months of real aging in distilled water or thermocycling) in adhesive restorative procedures that used experimental degradation-resistant monomers. It was observed that the monomers retrieved in the eligible studies were, in most cases, able to improve the bond strength after aging, in comparison to their conventional counterparts. Hence, the formulated hypothesis and research question in the present study was accepted. 

Hydrolytic degradation is considered one of the primary reasons for the biodegradation of resin-based materials, especially within the hybrid layer in dentin, contributing to the reduction in bond strength values over time, and consequently a short lifetime of resin-based composites [35,36]. This premature degradation occurs since most conventional monomers are methacrylate-based materials that contain ester bonds, highly prone to hydrolysis [37]. The overall water sorption/solubility of the polymeric adhesive mixture also decreases the stability of dental adhesives [38]. 

To overcome this problem, the incorporation of monomers with improved chemistry, such as ester-free monomers, in the organic matrix was suggested. As such, this systematic review included studies which proposed different monomers. These include secondary methacrylamides with a hydroxyl group, which are HEMAM, 1-methyl HEMAM, 2-methyl HEMAM, 2EM and 2dMM, multifunctional acrylamides such as BMAAPMA, TMAAEA, BAADA and DEBAAP and hybrid methacrylamides with methacrylate functionalities: HEMAM_Hy, 2dMM_Hy and 2EM_Hy. Despite their decreased reactivity and high hydrophilicity, which led to lower values for some mechanical parameters, experimental dental adhesive formulations which include alternatives to methacrylates, such as methacrylamides, show interesting features. Since they have more stable amide bonds than traditional ester bonds, they have demonstrated notable long-term dentin bonding stability, establishing themselves as a promising option for the design of hydrolytically stable adhesives [39,40]. 

Urushiol was used as a main monomer, as a result of its increased water resistance due to its chemical structure composed of a lengthy alkyl chain and a benzene ring [41,42], as well as antibacterial and antioxidant qualities [43], thus forming the basis for derived monomers analyzed in two of the studies included in this review.

In the study by Alkattan et al. in 2022, they reported the use of a multi-functional adhesive system with an experimental primer incorporating BMEP [44] and an adhesive containing EgMA in the formulation of a two-step self-etch system in order to provide an adhesive with long-lasting antibacterial activity that is chemically stable [31].

In 2019, Xu et al. synthesized an isocyanate-terminated urethane methacrylate precursor known as a collagen-reactive monomer (CRM) which has the ability to chemically bind to dentin collagen through covalent and hydrogen bonding in both wet and dry conditions [45]. Additionally, in 2021, Yu et al. designed an adhesive (CBA) based in the collagen-reactive monomer (CRM), with the aim that when used in conjunction with the carboxymethyl–chitosan (CMCS)-based extrafibrillar demineralization technique, comprising chelating chemicals, such as chitosan [46] and glycol chitosan-EDTA [47], it would enhance the dentin bonding strength. A commercially available CMCS was used, as its carboxylic group can increase the water solubility of chitosan [48]. All these monomers were included in the review and subsequent meta-analysis.

Turning to the immediate and aged bond strength results, different monomers showed distinct bond strength aging profiles. Methacrylamides and methacrylamide hybrids tested by Fugolin et al. (2020; 2021) showed substantially smaller bond strength differences after aging, when compared to the methacrylate controls. In the study by Fugolin et al., (2020) it was expected that the incorporation of difunctional molecules into the formulations would enhance the µTBS [26]. Between 48 h and 6 months, the µTBS decreased for all materials, and the HEMA_3 and two 2dMM compounds had a statistically significant decline, while HEMAM_2 showed the lowest bond strength reduction. Studies have revealed that the amides can create hydrogen bonds with particular collagen sites, which may have helped to fortify the substrate in some way [49]. Additionally, the amide bond is stronger than that of the methacrylates, as they form a double bond resulting from the nitrogen lone pairs’ donation of an electron to the N-C bond [50,51]. The presence of an oxygen atom in methacrylates, compared to the nitrogen atom in amides, leads to a greater susceptibility to hydrolysis [52]. This could explain the reason behind the bond stability after the aging of the methacrylamides. In comparison to HEMA_2 and the commercial control, with Adper Single Bond 2, all evaluated multi-acrylamides showed higher µTBS results at 24 h (the exception was BMAAPMA, which had statistically similar µTBS compared to the commercial control). In addition, after 6 months of storage, TMAAEA had negative values of µTBS, meaning that it had higher values of bond strength after aging than at the beginning;, therefore, the strength increased [28]. These results regarding TMAAEA were not expected, as bond strength tends to decrease over time. However, this can be explained by the fact that this monomer is trifunctional, while the remaining monomers in this study are difunctional, thus presenting a denser reticulated network, which makes it more hydrophobic, thus repelling hydrolytic attack [53]. Polymerization may have occurred gradually between the beginning of the test and the end, until all the monomers had converted into a polymeric network. In the presence of this extent of polymerization, the properties of the adhesive interface improve, as they contribute to greater cohesion at the adhesive interface [54].

With regard to the HEMA_αβ_mixture, the authors explained that the side-group replacements at the α- and β-carbons may have led to a poorly packed polymer network with reduced mechanical properties, which could account for the low µTBS [55]. At 6 months, HEMA derivatives (HEMA_1 and HEMA_αβ_mixture) showed unstable aged bond strength, which once again highlights the degradation resistance of the alternative methacrylamides.

Zhao et al. (2021) synthetized a novel photocurable urushiol derivative with a polymerization time described by the authors as immediate, for application in dental adhesives. This fact remains to be studied. Urushiol has mostly been used in dentistry up to this point due to its antibacterial characteristics, leaving its other properties unexplored. When creating etch-and-rinse adhesives, various concentrations of urushiol derivates were used in the replacement of Bis-GMA [30]. Quite interestingly, urushiol_65% showed the highest immediate bond strength values and the worst ones after aging, while all others followed a similar trend after aging. The authors combined urushiol with HEMA, a monomer known to aid the resin diffusion and interpenetration within demineralized dentin. There is likely an optimal concentration of urushiol/HEMA at which the immediate bond strength values are enhanced [56], although due to the high percentage of HEMA, the urushiol_65% formulation showed a marked decrease after aging. This may have also been potentiated by a lower extent of polymerization (a lower degree of conversion). Upon increasing the concentration of urushiol, more hydrophobicity was expected, thus leading to increased bond stability in this formulation. In another study by Zhao et al. (2022), primers of urushiol dissolved in DMSO or ethanol where synthetized. DMSO has been documented as a multifunctional solvent [57], entirely miscible in the majority of adhesive monomers [58] and able to modify the demineralized collagen structure [59]. It can also enhance bonding effectiveness under dry and wet conditions, allowing the increased penetration of adhesive into the exposed collagen matrix [60]. Because of the ability of DMSO to break down the self-associative tendency of water, it may also reduce the number of water molecules entrapped between the polymeric chains. The relative decrease in free water would eliminate or decrease the hydrolytic degradation of the adhesives [59]. The improvement in the hybrid layer caused by DMSO is still not entirely understood, even though a few mechanisms have been suggested to explain the higher resin-dentin bonding performance it causes [61]. According to some studies, ethanol stiffens demineralized collagen, maintaining the interfibrillar gaps in the collagen network, thus allowing the infiltration of hydrophobic resin monomers. As a result, it is possible to create a high-grade hydrophobic hybrid layer that has durability and bonding strength [62], compatible with better interpenetration during the adhesive procedure. This could correlate with the result of this study. Both strategies showed enhanced immediate bonding, as well as stability after aging.

There was no significant difference between the EgMA0, EgMA10 and EgMA20 treatments, as all had similar performance in both immediate and aged bond strengths. Based on a previous study by Rojo et al. (2006), it has been established that adding a polymerizable methacrylate group to the chemical structure of eugenol enables the eugenol derivative to take part in the free radical addition polymerization reaction, which is followed in resin-based dental materials [63]. This derivate, EgMA, which has been successfully included into glass-ionomer cements, resin composites and commercial adhesives for endodontic applications [64], demonstrates intrinsic antibacterial activity against a wide spectrum of oral bacteria produced by the immobilized agent [65].

According to the analysis, CMCS and CBA, together, presented favorable immediate μTBS values, regardless of whether a wet bonding or dry bonding technique was used, when compared to the other treatments of the same group. The wet bonding technique consists of keeping the dentin wet after conditioning, since after the dentin surface has dried, the interfibrillar spaces within the collagen network may collapse, preventing the penetration of adhesive resin monomers. On the other hand, the dry bonding technique is the opposite of the previous one, in which the dentin surface is dried, allowing for better removal of solvents and residual water [66]. In the study by Yu et al. (2021), the dry bonding technique was used since the antagonist technique is considered responsible for causing greater hydrolytic degradation due to the adhesive resin monomers that do not infiltrate the intrafibrillar spaces and due to the excess of water caused by this technique. The intrafibrillar spaces of the demineralized collagen matrix were shown to be difficult to infiltrate by the adhesive resin monomers [67]. Hence, the extrafibrillar demineralization technique was proposed, as it only leaves demineralized extrafibrillar gaps in the collagen matrix, enabling the infiltration of adhesive resin monomers and therefore creating an enhanced resin–dentin interface [68]. The CBA adhesive infiltrated the expanded collagen matrix created by the CMCS chelating demineralization technique and produced very favorable µTBS values. Dentin consists of organic and inorganic matter [69]; after removing the inorganic part, through acid etching, the gaps created are filled with adhesive resin [70]. According to a study by Gu et al. in 2019, the CMCS technique contributes to dentin hybridization, as it reduces the enzymatic hydrolysis mediated by MMPs in the collagen structure, promoting the stability of the resin–dentin interface.

Polymer networks for adhesive dentistry should ideally be made of insoluble materials with strong chemical stability. However, the majority of the monomers utilized in resin-based materials have the ability to absorb water and chemicals from the environment as well as release components into it [15,71]. It has also been demonstrated that the migration of water from hydrated dentin may result in the creation of water-filled channels within the polymer matrices [72]. This causes bacterial ingress and induces phase separation and hydrophilic–hydrophobic incompatibility, the leaching and release of unreacted monomers and marginal discoloration. [53]. A more hydrophilic adhesive has a higher water absorption rate, which causes the hybrid layer to hydrolyze more quickly [73]; thus, it is essential to create adhesives that have lower values of water sorption and water solubility.

Methacrylamides have the ability to form hydrogen bonds with water as both hydrogen-bond donors and acceptors (O-H and N-H dipoles, respectively). Hence, methacrylamides are more hydrophilic than methacrylates, due to the amide’s nitrogen atoms’ higher electronegativity when compared to methacrylate’s oxygen atom [52]. Therefore, in the study by Fugolin et al., 2020, to lessen the hydrophilicity of the secondary methacrylamides, the methacrylate functionality was added to those compounds [26]. As a result, the methacrylate–methacrylamide hybrids (HEMAM Hy, 2EM Hy and 2dMM Hy) showed a substantial reduction in water sorption. The alpha-substituted methacrylamides (2EM and 2dMM) demonstrated positive solubility results, indicating a higher degree of mass loss due to the leaching out of unreacted monomers. The diacrylamide DEBAAP presented low values of water sorption that could be explained by the high hydrophobicity confirmed through the values of the octanol/water partition coefficient (logP) [74]. The logP value is used as a parameter to determine the hydrophobic or hydrophilic character of a compound, with lower or negative values referring to more hydrophilic compounds [75]. Regarding water solubility, some groups showed negative values, according to Fugolin et al., 2020, indicating hygroscopic expansion due to time [28]. This leads to water being retained within the polymer network, which compensates for the volume of unreacted monomer lost. All monomers with negative values retained water and expanded their mass, that is, they did not show solubility.

The formulation containing urushiol_70% had the lowest water sorption values and similar values of solubility when compared to the experimental adhesives of the same group. This could be explained by the fact that they have in their composition an urushiol derivate that is made up of hydrophobic groups discussed above. In this study, the adhesive’s derivative content was raised from 55% to 70%. The W*sp* and W*sl* of the experimental adhesives steadily and considerably reduced to a level lower than that of Adper Single Bond 2, possibly because of the increased concentration of this monomer. This monomer may have contributed to an increased cross-linking of the polymer network, higher than in the commercial control group, which could have a significant impact on water dynamics, favorable to its reduced degradation [30].

Using EgMA in the formulation of the novel adhesives could reduce the water sorption values. All three experimental adhesives, which are EgMA0, EgMA10 and EgMA20, also demonstrated similar solubility. This could be due to EgMA’s hydrophobic properties and its capacity to create slightly cross-linked structures. Adhesives’ hydrophobicity significantly increases, which in turn leads to a decrease in their water sorption values [76].

Statistical analysis of bond failure modes was not included in the present review, as it was not the scope of the quantitative synthesis planned for this study, as published in the PROSPERO protocol (referenced under 2. Materials and Methods). Furthermore, the majority of the studies that were included did not report bond failure modes, which is also why they were judged as having incomplete outcome data. Thus, a thorough synthesis and comparison of this information across the seven studies would not have been possible.

The development and testing of degradation-resistant and hydrolytically stable monomers is one of the key solutions to overcome aging effects in unstable substrates. However, few studies focusing on this can be found in the modern literature, as was seen in the present systematic review. Even in the studies that are available, there is a need to improve and standardize the tests/assays and their reporting, so as to enhance scientific quality. The following factors should be standardized in future studies—a priori sample size calculation, adequate standardized testing procedures, outcomes and better outcome reporting. Most of the studies presented showed the insufficient reporting of standardized testing procedures and outcomes. This was mainly related to having tested microtensile bond strength without referencing or complying with the latest ADM guidance [77], which advises aspects that were lacking, such as bond failure mode, the recommended sample size for testing or equipment handling. Failing to provide sufficient methodological data hampers bias judgement. In the future, this bias minimization strategy will allow the publication of leading reference laboratory studies in the field.

## 5. Conclusions

The use of methacrylamides and methacrylamide–methacrylate hybrid monomers in the formulation of dental adhesives has been shown to have better long-term bond strength results when compared with methacrylates. Likewise, urushiol primers dissolved in DMSO or ethanol obtained some of the best immediate and aged bond strength results, as did the new urushiol derivative that was synthesized. Regarding the values of water sorption and water solubility, in general, methacrylamides, urushiol compounds and EgMA-based adhesives obtained better values than the respective control groups. As such, most of these experimental monomers show very favorable and interesting degradation and adhesion profiles, for inclusion in new generations of adhesives that intend to fill the present flaws found in the hybrid adhesive layer.

It was also important to determine that it is essential to proceed with the standardization of laboratory studies for the design of new dental materials in order to obtain comparable results and thus improve the scientific evidence currently available.

## Figures and Tables

**Table 1:** Search strategy used in each database to retrieve full-text papers.

Database	Search Strategy
PubMed/Medline	(“degradation resistant” OR “ester free” OR “hydroly* resistan*” OR “ether based” OR “hydrolytic stability” OR “methacrylamide based” OR “ether linkage” OR “ether group”) AND (resin OR adhes* OR “dentin bonding agent” OR “dental polymer” OR “dental adhes*”) AND (dentin* OR “hybrid layer”) AND (bond* OR “bond strength” OR “tensile strength” OR “bond interface” OR “aging”)
Scopus	TITLE-ABS-KEY ((“degradation resistant”) OR (“ester free”) OR (“hydroly* resistan*”) OR (“ether based”) OR (“hydrolytic stability”) OR (“methacrylamide based”) OR (“ether linkage”) OR (“ether group”)) AND ((“resin”) OR (adhes*) OR (“dentin bonding agent”) OR (“dental polymer”) OR (“dental adhes*”)) AND ((dentin*) OR (“hybrid layer”)) AND ((bond*) OR (“bond strength”) OR (“tensile strength”) OR (“bond interface”) OR (“aging”))
SciELO	((degradation resistant) OR (ester free) OR (hydroly* resistan*) OR (ether based) OR (hydrolytic stability) OR (methacrylamide based) OR (ether linkage) OR (ether group)) AND ((resin) OR (adhes*) OR (dentin bonding agent) OR (dental polymer) OR (dental adhes*)) AND ((dentin*) OR (hybrid layer)) AND ((bond*) OR (bond strength) OR (tensile strength) OR (bond interface) OR (aging))
EMBASE	((“degradation resistant” or “ester free” or “hydroly* resistan*” or “ether based” or “hydrolytic stability” or “methacrylamide based” or “ether linkage” or “ether group”) and (resin or adhes* or “dentin bonding agent” or “dental polymer” or “dental adhes*”) and (dentin* or “hybrid layer”) and (bond* or “bond strength” or “tensile strength” or “bond interface” or “aging”))

**Table 2:** Systematic review table summarizing the study characteristics: author/date, country of the study, sample size, experimental monomer, experimental groups, aging method, aging period and final conclusions.

Author	Country	Sample Size	Experimental Monomer	Experimental Groups	Aging Method	Aging Period	Conclusion
Fugolin et al. (2020) [26]	United States	*n* = 6	2-hydroxyethyl methacrylate (HEMA) N-hydroxyethyl methacrylamide (HEMAM) N-hydroxyethyl methacrylamide with Methacrylate functionality (HEMAM Hy) HEMAM modified with methyl substituents on the first (alpha) carbon (2dMM) 2dMM with methacrylate functionalities (2dMM Hy) HEMAM modified with ethyl substituents on the first (alpha) carbon (2EM) 2EM with methacrylate functionalities (2EM Hy)	Control group: Adper Single Bond [ER] (3M ESPE) Exp_1: Experimental adhesive with HEMA Exp_2: Experimental adhesive with HEMAM Exp_3: Experimental adhesive with HEMAM Hy Exp_4: Experimental adhesive with 2dMM Exp_5: Experimental adhesive with 2dMM Hy	Water storage	6 months	The hybrid versions methacrylate/methacrylamide showed lower values of water sorption and solubility. The µTBS values between 48 h and 6 months were reduced only for the HEMA and both 2dMM materials.
Fugolin et al. (2020) [28]	United States	*n* = 6	N, N-Diethyl-1,3-bis(acrylamido)propane (DEBAAP) N, N-Bis[(3-methylaminoacryl) propyl] methylamine (BMAAPMA) Tris[(2-methylaminoacryl) ethyl] amine (TMAAEA) N, N’-bis(acrylamido) 1,4-diazepane (BAADA)	Control group: Adper Single Bond 2 [ER] (3M ESPE) Exp_1: Experimental adhesive with 2-Hydroxyethyl methacrylate (HEMA) Exp_2: Experimental adhesive with DEBAAP Exp_3: Experimental adhesive with BMAAPMA Exp_4: Experimental adhesive with TMAAEA Exp_5: Experimental adhesive with BAADA	Water storage (distilled water)	6 months	The acrylamide-containing materials presented enhanced interfacial bond strength stability compared to the methacrylate control and demonstrated higher water sorption.
Fugolin et al. (2021) [32]	United States	*n* = 6	2-hydroxyethyl methacrylamide (HEMAM) 2-hydroxy-1-methylethyl methacrylamide (1-methyl HEMAM) 2-hydroxy-2-methylethyl methacrylamide (2-methyl HEMAM)	Control group: Adper Single Bond [ER] (3M ESPE, St. Paul, MN, USA) Exp_1: Experimental adhesive with 2-hydroxyethylmethacrylate (HEMA) Exp_2: Experimental adhesive with 72% β-substituted 2-hydroxy-2-methylethyl methacrylamide and 28% α-substituted 2-hydroxy 1-methylethyl methacrylate (HEMA α-β mixture) Exp_3: Experimental adhesive with HEMAM Exp_4: Experimental adhesive with 1-methyl HEMAM Exp_5: Experimental adhesive with 2-methyl HEMAM	Water storage (distilled water)	6 months	Methacrylamides presented increased resistance to hydrolysis and higher bond strengths than the analogous methacrylates.
Yu et al. (2021) [33]	China	*n* = 10	Adhesive based on collagen reactive monomer (CBA)	Control group_1: Solobond M [ER] (VOCO) + phosphoric acid + Dry Bonding technique Control group_2: Solobond M [ER] (VOCO) + phosphoric acid + Wet Bonding technique Control goup_3: Solobond M [ER] (VOCO) + 1 wt% of carboxymethyl chitosan (CMCS) + Dry Bonding technique Control group_4: SolobondM [ER] (VOCO) + 1 wt% of carboxymethyl chitosan (CMCS) + Wet Bonding technique Exp_1: Experimental adhesive with CBA + phosphoric acid + Dry Bonding technique Exp_2: Experimental adhesive with CBA + phosphoric acid + Wet Bonding technique Exp_3: Experimental adhesive with CBA + 1 wt% of carboxymethyl chitosan (CMCS) + Dry Bonding technique Exp_4: Experimental adhesive with CBA + 1 wt% of carboxymethyl chitosan (CMCS) + Wet Bonding technique	Thermocycling	30,000 cycles	The bonding scheme containing CMCS and CBA achieved promising dentin bonding strength and durability when used with the dry-bonding technique.
Zhao et al. (2021) [30]	China	*n* = 9	Urushiol derivatives	Control group: Adper Single Bond 2 [ER] (3M, St. Paul, MN, USA). Exp_1: Experimental adhesive with 55 wt% of urushiol derivative and 45 wt% of HEMA Exp_2: Experimental adhesive with 60 wt% of urushiol derivative and 40 wt% of HEMA Exp_3: Experimental adhesive with 65 wt% of urushiol derivative and 35 wt% of HEMA Exp_4: Experimental adhesive with 70 wt% of urushiol derivative and 30 wt% of HEMA	Thermocycling	5000 cycles	The water sorption/solubility of the novel urushiol derivative monomer was significantly lower, whilst the µTBS were higher compared to the control group.
Alkattan et al. (2022) [31]	United Kingdom	*n* = 6	Eugenyl methacrylate (EgMA)	Control group: Experimental adhesive without Eugenyl methacrylate (EgMA0) Exp_1: Experimental adhesive with eugenyl methacrylate at concentration of 10 wt% (EgMA10) Exp_2: Experimental adhesive with eugenyl methacrylate at concentration of 20 wt% (EgMA20)	Water storage (distilled water)	6 months	Higher concentrations of EgMA in the adhesive significantly demonstrated lower water sorption and solubility and improved bond durability after aging.
Zhao et al. (2022) [34]	China	*n* = 6	Urushiol derivatives	Control group: Adper Single Bond 2 [ER] (3M, St. Paul) Exp_1: Experimental primer with Ethanol Exp_2: Experimental primer with urushiol at concentration of 0.1 wt% dissolved in ethanol (Ethanol Urushiol 0.1%) Exp_3: Experimental primer with urushiol at concentration of 0.5 wt% dissolved in ethanol (Ethanol Urushiol 0.5%) Exp_4: Experimental primer with urushiol at concentration of 0.7 wt% dissolved in ethanol (Ethanol Urushiol 0.7%) Exp_5: Experimental primer with urushiol at concentration of 1 wt% dissolved in ethanol (Ethanol Urushiol 1%) Exp_6: Experimental primer with DMSO Exp_7: Experimental primer with urushiol at concentration of 0.1 wt% dissolved in DMSO (DMSO Urushiol 0.1%) Exp_8: Experimental primer with urushiol at concentration of 0.5 wt% dissolved in DMSO (DMSO Urushiol 0.5%) Exp_9: Experimental primer with urushiol at concentration of 0.7 wt% dissolved in DMSO (DMSO Urushiol 0.7%) Exp_10: Experimental primer with urushiol at concentration of 1 wt% dissolved in DMSO (DMSO Urushiol 1%)	Thermocycling	5000 cycles	The application of urushiol primer shoved improved bonding strength, particularly after aging.
ER = Etch-and-rinse; µTBS = microtensile bond strength							

**Table 3:** RoB analysis for the 7 studies included in this SR, shown in Table 2.

Author	D1: Bias in Planning and Allocation			D2: Bias in Specimen Preparation		D3: Bias in Outcome Assessment		D4: Bias in Data Treatment and Reporting	
	Control Group	Sample Size Calculation	Correct Randomization of Samples	Identical Experimental Conditions	Standardization of Samples and Materials	Adequate and Standardized Testing Procedures/ Outcomes	Blinding of the Testing Operator	Appropriate Statistical Analysis	Correct Reporting of Outcomes
Fugolin et al. (2020) [26]	Reported	Not reported	Not reported	Reported	Reported	Insufficiently reported	Not reported	Insufficiently reported	Insufficiently reported
Fugolin et al. (2020) [28]	Reported	Not reported	Not reported	Reported	Reported	Insufficiently reported	Not reported	Insufficiently reported	Insufficiently reported
Fugolin et al. (2021) [32]	Reported	Not reported	Not reported	Reported	Reported	Insufficiently reported	Not reported	Insufficiently reported	Insufficiently reported
Yu et al. (2021) [33]	Reported	Not reported	Not reported	Insufficiently reported	Reported	Insufficiently reported	Not reported	Reported	Insufficiently reported
Zhao et al. (2021) [30]	Reported	Not reported	Insufficiently reported	Insufficiently reported	Reported	Insufficiently reported	Not reported	Reported	Reported
Alkattan et al. (2022) [30]	Reported	Not reported	Not reported	Insufficiently reported	Reported	Insufficiently reported	Not reported	Reported	Reported
Zhao et al. (2022) [34]	Reported	Not reported	Insufficiently reported	Insufficiently reported	Insufficiently reported	Not reported	Not reported	Insufficiently reported	Insufficiently reported

## Data Availability

Not applicable.
